# Conditional Expression of Smad7 in Pancreatic β Cells Disrupts TGF-β Signaling and Induces Reversible Diabetes Mellitus

**DOI:** 10.1371/journal.pbio.0040039

**Published:** 2006-01-31

**Authors:** Nora G Smart, Åsa A Apelqvist, Xueying Gu, Erin B Harmon, James N Topper, Raymond J MacDonald, Seung K Kim

**Affiliations:** **1**Department of Developmental Biology, Stanford University School of Medicine, Stanford, California, United States of America; **2**Division of Cardiology, Department of Medicine, Stanford University School of Medicine, Stanford, California, United States of America; **3**Department of Molecular Biology and Oncology, University of Texas at Southwestern, Dallas, Texas, United States of America; **4**Division of Oncology, Department of Medicine, Stanford University School of Medicine, Stanford, California, United States of America; }}Rahilly, University of CambridgeUnited Kingdom

## Abstract

Identification of signaling pathways that maintain and promote adult pancreatic islet functions will accelerate our understanding of organogenesis and improve strategies for treating diseases like diabetes mellitus. Previous work has implicated transforming growth factor-β (TGF-β) signaling as an important regulator of pancreatic islet development, but has not established whether this signaling pathway is required for essential islet functions in the adult pancreas. Here we describe a conditional system for expressing Smad7, a potent inhibitor of TGF-β signaling, to identify distinct roles for this pathway in adult and embryonic β cells. Smad7 expression in *Pdx1* ^+^ embryonic pancreas cells resulted in striking embryonic β cell hypoplasia and neonatal lethality. Conditional expression of Smad7 in adult *Pdx1* ^+^ cells reduced detectable β cell expression of MafA, menin, and other factors that regulate β cell function. Reduced pancreatic insulin content and hypoinsulinemia produced overt diabetes that was fully reversed upon resumption of islet TGF-β signaling. Thus, our studies reveal that TGF-β signaling is crucial for establishing and maintaining defining features of mature pancreatic β cells.

## Abstract 2

TGF-β signaling is known to regulate the development of pancreatic β cells; here the authors show that TGF-β is also required for the maintenance of β cell identity in the adult.

## Introduction

A major goal in studies of pancreatic islet development and diabetes mellitus is the identification of signaling pathways that maintain adult pancreatic islet functions. Pancreatic islets are comprised of endocrine cells that secrete hormones including insulin (β cells), glucagon (α cells), somatostatin (δ cells), pancreatic polypeptide (PP cells), and ghrelin ( cells). Disrupted transforming growth factor-β (TGF-β) signaling impairs embryonic pancreatic β cell differentiation [ 1– 3], suggesting essential roles for this pathway in islet development. However, redundant TGF-β signaling activities and pleiotropism from germline mutations producing postnatal lethality have limited conclusions about the role of this signaling pathway in adult islets [ 3– 5]. Thus, it remains unclear if TGF-β signaling disruption may impair islet function, leading to pathogenesis of diseases like diabetes.

Multiple components of the TGF-β signaling pathways are expressed in the embryonic and adult pancreas, consistent with the hypothesis that TGF-β activities regulate pancreas development and function [ 3, 6– 9]. The developing and adult pancreas expresses multiple TGF-β ligands, like TGF-β1, activins, and bone morphogenetic proteins (BMPs), which are known to bind heteromeric receptor complexes comprised of membrane-associated type I and type II receptor kinases. Ligand activation of these receptors triggers phosphorylation of intracellular proteins called receptor-regulated Smads (R-Smads), which form heteromeric complexes with Smad 4. The activated Smad complexes translocate to the nucleus, where they regulate the transcription of target genes. R-Smads 1, 5, and 8 transduce BMP signaling, while R-Smads 2 and 3 are thought to mediate TGF-β and activin signaling [ 10]. The inhibitory Smads, Smad6 and Smad7, block TGF-β signaling by blocking R-Smad phosphorylation and may also target TGF-β receptors for degradation [ 10– 13].

Prior in vivo and in vitro studies have identified TGF-β signaling factors necessary for specific steps of pancreatic development. β cell differentiation appears to be particularly sensitive to mutations affecting TGF-β signaling components, including the activin receptors ActRIIA and ActRIIB, Smad2, and the ligand growth/differentiation factor 11 (Gdf11 [ 2– 3]). Recently, we showed that Gdf11 is required for development of the appropriate number of endocrine precursor cells expressing the bHLH factor neurogenin3, and for the differentiation of embryonic β cell precursors into mature insulin-producing cells during pancreas development. These prior studies suggested that TGF-β signaling was required for β cell differentiation, but the islet cell defects revealed in these studies were modest and likely reflected redundant TGF-β signaling activities. Mutations in single genes or compound mutation combinations affecting TGF-β components also impaired glucose tolerance in adult animals but did not produce overt diabetes [ 2, 3]. Based on the mild physiologic effects following TGF-β signaling disruption in these and other studies [ 14, 15], it was not possible to conclude unambiguously that TGF-β signaling was required to maintain adult islet cell function. Moreover, it was not known if the mildly impaired glucose regulation previously observed in these studies of adult germline mutants provided evidence for essential TGF-β signaling roles in adult islets, or merely reflected the late, secondary consequences of impaired embryonic endocrine development.

Several classes of ligands comprise the TGF-β superfamily, including activins, BMPs, and TGF-β isoforms, and Smad7 is a potent in vivo inhibitor of signaling by each of these ligand groups [ 11, 12, 16]. We generated transgenic mouse lines that permit conditional expression of Smad7 in Pdx1-expressing cells in the embryonic or adult pancreas. Pdx1 is expressed in all pancreatic progenitor cells and becomes largely restricted to β cells in islets by the end of embryonic development [ 17– 19]. Disruption of TGF-β signaling by Smad7 revealed crucial in vivo roles for the TGF-β pathway in pancreatic β cell development, and demonstrated that this pathway is essential for maintaining characteristic features and hallmark functions of adult β cells.

## Results

### Conditional Expression of Smad7 in Embryonic Pdx1 ^+^ Cells Disrupts TGF-β Signaling, Foregut Development, and Endocrine Cell Differentiation

**Figure 1:**
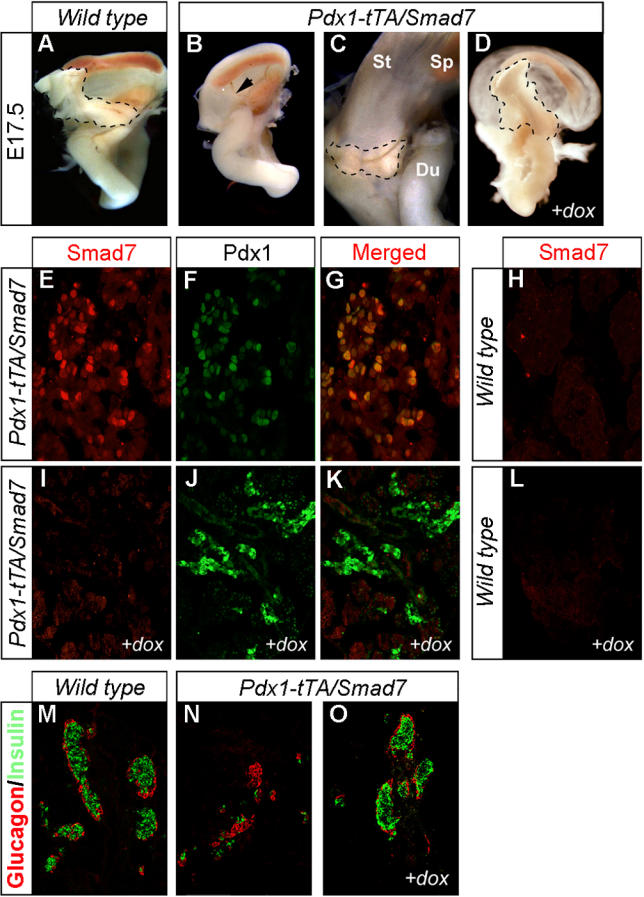
Conditional Smad7 Expression in Embryonic Pdx1 ^+^ Cells Leads to Pancreatic Malformation (A–D) Whole-mounted abdominal organs from E17.5 embryos with the indicated genotypes. (A–C) Views of the pancreas (outlined by dashes) at E17.5 in wild-type and bitransgenic *Pdx1-tTA/Smad7* mice. (C) shows a magnified view of the pancreatic region marked by the arrow in (B). Adjacent organs marked in (C): St, stomach; Sp, spleen; Du, duodenum. (D) Bitransgenic *Pdx1-tTA/Smad7* embryos from doxycycline-fed mothers (+ *dox*) develop a morphologically normal pancreas. (E–L) Smad7 (red) or Pdx1 (green) expression detected by immunohistochemistry in the pancreas of E14.5 mice with the indicated genotypes. (M–O) Immunohistochemical detection of glucagon ^+^ (red) and insulin ^+^ (green) cells at P1 in mice with the indicated genotypes. (O) Bitransgenic *Pdx1-tTA/Smad7* embryos from doxycycline-fed mothers (+ *dox*) develop normal-appearing islets. Original magnification is 4× for (A–D), 63× for (E–H), and 20× for (I–O).

To permit studies of phenotypes following disruption of pancreatic TGF-β signaling, we generated transgenic mouse lines that permitted conditional expression of *Smad7,* a potent inhibitor of signaling by TGF-β ligands, including activin, BMPs, and TGF-β isoforms [ 11, 12]. Mice harboring a transgene encoding Smad7 adjacent to a tetracycline response element (TRE) containing a *cis-*acting heptameric tetracycline-regulated transactivator (tTA)-binding site and a minimal promoter were created (hereafter called *Smad7* mice; see Materials and Methods) [ 20, 21]. *Smad7* mice were bred with *Pdx1-tTA* mice in which the tTa replaces the coding region of *Pdx1,* a gene expressed in pancreatic progenitor cells as well as in adult β cells [ 17– 19, 22]. Thus, in *Pdx1-tTA* mice, the tTA is expressed in the spatial and temporal pattern of the endogenous *Pdx1* gene. tTA-induced expression of TRE-Smad7 is repressed upon administration of the drug doxycycline, a tetracycline analog ( Figure 1). Bitransgenic *Pdx1-tTA/Smad7* progeny from this intercross were obtained at the expected frequency from more than 40 litters.

In 23 bitransgenic embryos raised in the absence of doxycycline, we observed severe pancreatic malformation and lethality at postnatal day (P)1 ( Figure 1A– 1D). We also observed malformations of the spleen and duodenum, a pattern of organ defects consistent with the established expression of *Pdx1*- *tTA* in the posterior foregut (unpublished data) [ 22]. In the absence of doxycycline in *Pdx1-tTA/Smad7* mice, tTA activated expression of Smad7 in Pdx1 ^+^ cells of the embryonic pancreas ( Figure 1E– 1G). Bitransgenic embryos showed a marked decrease in all phosphorylated R-Smads ( Figure S1), which is consistent with the conclusion that Smad7 expression in our system reduced signaling through TGF-β pathways.

In *Pdx1-tTA/Smad7* embryos, we observed an 85%–90% reduction of β cells at P1, compared to control littermates ( Figure 1M and 1N; Figure S1). In contrast, the number of glucagon ^+^ cells was increased, and these formed anomalous homogeneous clusters in these mice ( Figure 1M– 1N; Figure S1). These defects were observed as early as E15.5 (unpublished data) and were similar to phenotypes we have observed in mice with germline mutations in specific TGF-β components [ 2, 3]. We did not observe significant changes in the proportion of cells expressing other islet hormones, including pancreatic polypeptide ( *p* = 0.16), somatostatin ( *p* = 0.24), and ghrelin ( *p* = 0.82). No changes were detected in cell proliferation or apoptosis in *Pdx1-tTA/Smad7* mice at E15.5 and E17.5, as assessed by quantification of Ki67 or TdT-mediated dUTP nick-end labeling ( Figure S1 and unpublished data), suggesting that the observed phenotypes were due to cell differentiation defects. However, since apoptosis in β cells can be difficult to discern [ 23], we cannot strictly rule out the contribution of programmed cell death to the phenotypes observed in embryonic *Pdx1*-tTA/ *Smad7* mice.

To test whether doxycycline administration could prevent TRE- *Smad7* expression, we fed doxycycline to females throughout pregnancy and examined *Pdx1-tTA/Smad7* progeny. Doxycycline administration to mothers prevented TRE- *Smad7* expression in the embryonic pancreas; only low levels of endogenous Smad7 expression were detected in bitransgenic *Pdx1-tTA/Smad7* embryos, similar to wild-type controls ( Figure 1H and 1L). Bitransgenic *Pdx1-tTA/Smad7* mice from doxycycline-fed mothers had normal pancreas ( Figure 1D) and islet development ( Figure 1O). When maintained on doxycycline as adults, *Pdx1-tTA/Smad7* animals had normal glucose regulation (see below). Thus, adult *Pdx1-tTA/Smad7* mice provided an opportunity to test the effects of induced *Smad7* expression on β cell functions in vivo, independent of embryonic defects.

### Conditional Disruption of TGF-β Signaling in Adult Pancreatic Islets

**Figure 2:**
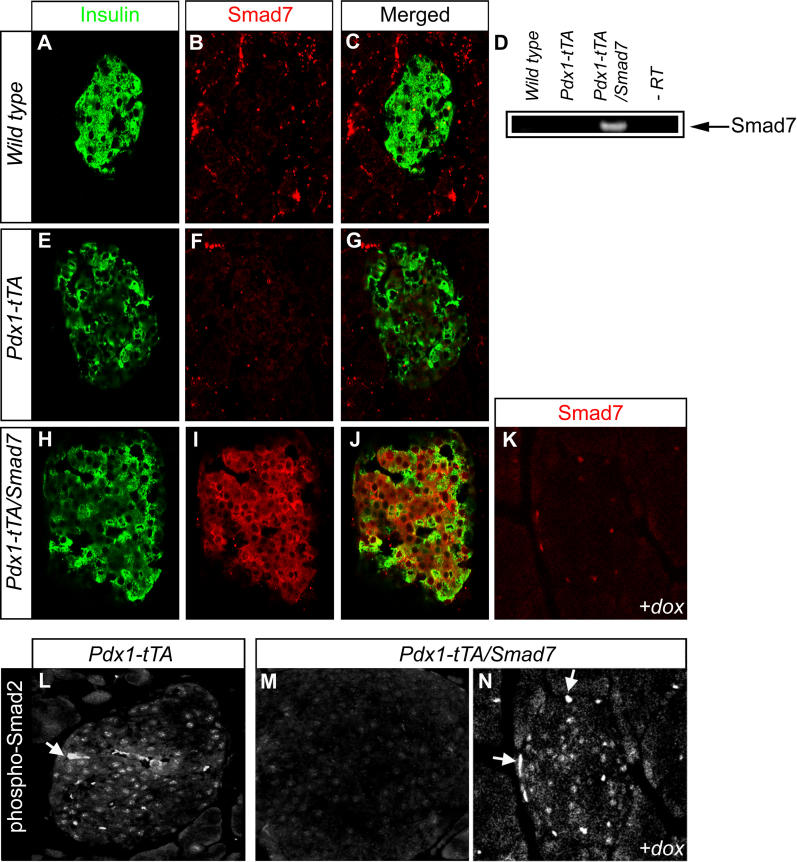
Doxycycline-Dependent Smad7 Expression and TGF-β Signaling Inhibition in *Pdx1-tTA/Smad7* Adult Islets Data are from tissues of male mice aged 4–5 mo. (A–C and E–J) Smad7 expression by β cells in wild-type and *Pdx1-tTA* controls is undetectable while immunohistochemical studies demonstrate Smad7 expression (red) in insulin-expressing β cells (green) of bitransgenic *Pdx1-tTA, TRE-Smad7* adults. (D) RT-PCR studies of purified pancreatic islets confirm increased accumulation of *Smad7* mRNA (arrow) in the islets of bitransgenic *Pdx1-tTA/Smad7* adults. (K) In bitransgenic animals induced to overexpress Smad7 then treated with doxycycline ( *+ dox*), Smad7 expression returns to wild-type levels. (L–N) Immunohistochemical detection of phospho-Smad2 (white). (L and M) Compared to *Pdx1-tTA* β cells, accumulation of phospho-Smad2 is decreased in β cells of bitransgenic *Pdx1-tTA/Smad7* adults. (N) phospho-Smad2 expression in β cells of *Pdx1-tTA/Smad7* animals treated with doxycycline (+ *dox*) returns to normal. In (L, N), arrows point to autofluorescent blood cells. Original magnification of all micrographs is 63×.

Next, we tested whether our bitransgenic mice could be used to express Smad7 conditionally in adult pancreatic islets ( Figure 2). In control adults and in bitransgenic *Pdx1-tTA/Smad7* animals continuously exposed to doxycycline from conception, islet expression of Smad7 was not detected by immunohistochemical staining or by RT-PCR ( Figure 2A–K, and unpublished data). The presence of active TGF-β signaling in these islets was indicated by detection of phosphorylated Smad2 (phospho-Smad2; Figure 2L and 2N) and phosphorylated Smad1/5/8 (unpublished data) using polyclonal antisera. Smad7 mRNA in islets and Smad7 protein in β cells were clearly detected in bitransgenic *Pdx1-tTA/Smad7* mice after doxycycline removal ( Figure 2D and 2H– 2J). Corresponding with Smad7 expression, we observed reduction of phospho-Smads from the majority of islet cells ( Figure 2M, unpublished data), consistent with the interpretation that Smad7 misexpression resulted in disrupted TGF-β signal transduction.

In a subset of bitransgenic *Pdx1-tTA/Smad7* mice re-exposed to doxycycline after a doxycycline-free interval of several weeks (see Materials and Methods), Smad7 expression in islets was extinguished ( Figure 2K), corresponding with the reappearance of detectable phospho-Smad2 ( Figure 2N). Thus, β cells in bitransgenic *Pdx1-tTA/Smad7* mice remained competent for endogenous pancreatic signals, and the reappearance of phospho-Smad2 suggests that one or more of these signals is a TGF-β ligand. These results demonstrate our ability to express Smad7 and impair TGF-β signaling conditionally in adult pancreatic islets.

### Smad7 Expression in Adult Islets Induces Overt Diabetes

**Figure 3:**
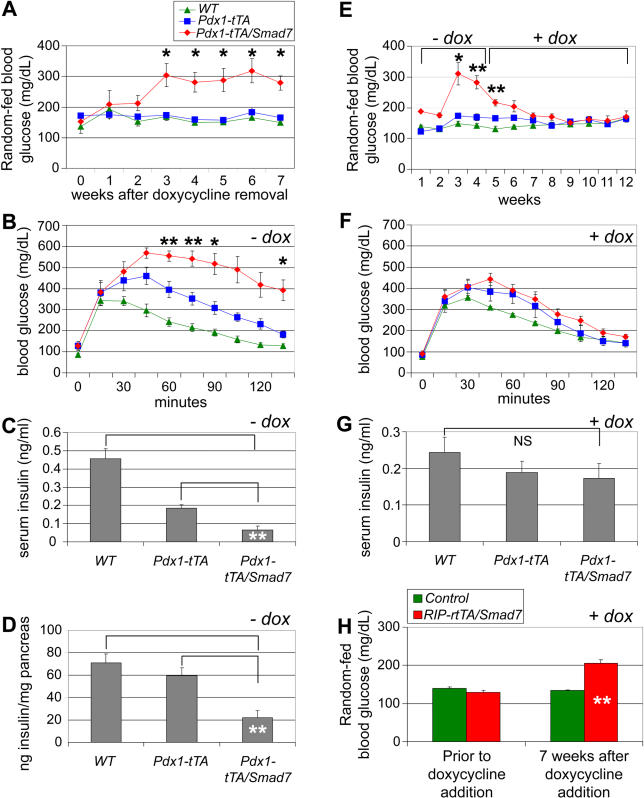
Diabetes and Reduced Pancreatic Insulin Production following Smad7 Expression in Pancreatic β Cells Data are presented as the average ± SEM. Data are from ten litters with at least four mice per genotype. *p-*Values and statistical significance in (A–G) reflects comparison of bitransgenic mice to *Pdx1-tTA* controls, unless otherwise indicated. (A) Random-fed blood glucose levels of male mice with indicated genotypes. Similar data were obtained for female littermates (unpublished data). (B) Intraperitoneal glucose challenge of male mice (8–10 wk old) with the indicated genotypes after a 14-h overnight fast. (C) Serum insulin from male mice with the indicated genotypes during random feeding. Mice were 8–10 wk old. (D) Total pancreatic insulin levels in 10- to 12-wk-old mice with the indicated genotypes. Insulin levels are expressed as ng/mg pancreas protein. (E–G) Reversal of overt diabetes mellitus and impaired insulin levels by treating bitransgenic *Pdx1-tTA/Smad7* mice with doxycycline. (E) Blood glucose levels during random feeding following exposure of mice with indicated genotypes and ages (weeks after birth) to doxycyline. (F) Intraperitoneal glucose challenge following a 14-h overnight fast in 12- to 14-wk-old mice with the indicated genotypes after 5 wk of continuous doxycycline exposure. (G) Serum insulin during random feeding from 12- to 14-wk-old mice with the indicated genotypes after 5 wk of continuous doxycycline exposure. (H) Induction of Smad7 by doxycycline in insulin ^+^ cells using *RIP-rtTA/Smad7* animals also yields mice with elevated levels of random-fed blood glucose. For all panels: * *p* < 0.05; ** *p* < 0.01.

Within 2–3 wk after doxycycline removal, Smad7 expression in *Pdx1-tTA/Smad7* islets was detected ( Figure 2H–J), and bitransgenic mice ( Figure 3) developed significant hyperglycemia during random feeding ( Figure 3A) and showed impaired glucose tolerance ( Figure 3B). Control *Pdx1-tTA* littermates, which are heterozygous for a null mutation in *Pdx1,* also had impaired glucose tolerance ( Figure 3B), but never developed overt diabetes ( Figure 3A), consistent with prior reports [ 22, 24, 25]. Moreover, impaired glucose tolerance in *Pdx1-tTA* mice was significantly less severe than that observed in bitransgenic *Pdx1-tTA/Smad7* mice. Thus, Smad7 expression in *Pdx1-*expressing islets resulted in overt diabetes, suggesting that Smad7 inhibits processes necessary for normal β cell function. To test this possibility further, and to assess the contribution of *Pdx1* heterozygosity to diabetes in these bitransgenic mice, we also studied glucose regulation in mice simultaneously harboring the *RIP-rtTA* and TRE- *Smad7* transgenes. The reverse tetracycline transactivator (rtTA) is expressed from rat insulin promoter (RIP) sequences in the *RIP-rtTA* transgene (see Materials and Methods). Exposure to doxycycline activates the rtTA product, resulting in Smad7 expression. In bitransgenic *RIP-rtTA*/ *Smad7* mice, in which both copies of *Pdx1* are wild-type, doxycycline exposure resulted in average blood glucose values >200 mg/dl during random feeding ( Figure 3H). Thus, independent of *Pdx1* heterozygosity, conditional expression of *Smad7* in islets produced overt diabetes.

**Figure 4:**
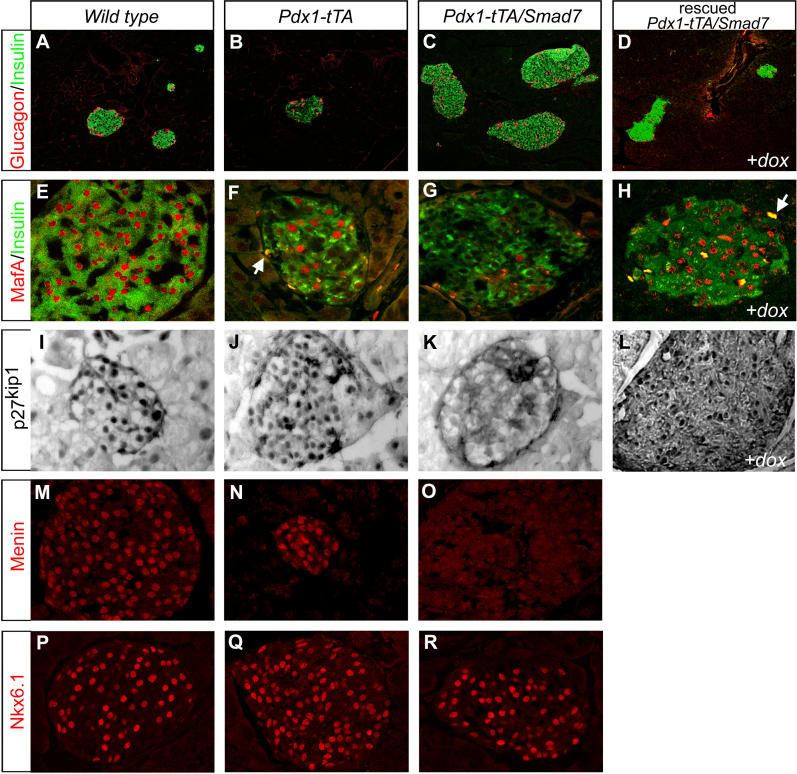
Conditional Smad7 Expression Alters Expression of Factors Important for Maintaining Adult β Cells Data are from tissues of male mice aged 4–5 mo. (A–C) Immunofluorescent detection of insulin ^+^ β cells (green) and glucagon ^+^ β cells (red) in wild-type, *Pdx1-tTA*, and bitransgenic *Pdx1-tTA/Smad7* mice. (C) Islet enlargement was detected in some bitransgenic *Pdx1-tTA/Smad7* mice following doxycycline withdrawal. (D) Islet morphology in paraffin sections appeared normal in bitransgenic *Pdx1-tTA/Smad7* animals 5 wk after readministration of doxycycline, as described in Materials and Methods. (E–H) Smad7 alters expression of MafA (red) in β cells expressing insulin (green). (F and G) The number of MafA-expressing β cells in bitransgenic *Pdx1-tTA/Smad7* was reduced compared to controls ( *n* = 3). (E and F) *Pdx1-tTA* animals also showed a mild decrease in numbers of MafA expressing islet cells relative to wild-type controls. (H) After diabetic bitransgenic *Pdx1-tTA/Smad7* animals were readministered doxycycline (+ *dox*), islet MafA expression appeared normal. Staining was performed on paraffin sections. (I–K, M–O) The expression of nuclear p27 ^kip1^ (black, I–K) and Menin (red, M–O) was reduced in adult *Pdx1-tTA/Smad7* animals compared to wild-type and *Pdx1-tTA* controls ( *n* = 5). (L) After diabetic bitransgenic *Pdx1-tTA/Smad7* animals were readministered doxycycline (+ *dox*), p27 ^kip1^ expression appeared relatively normal. (P–R) Nkx6.1 expression (red) was unchanged in *Pdx1-tTA/Smad7* animals. Arrows in (H) and (F) mark autofluorescent blood cells. Original magnification is 20× (A–D, I–L), and 63× (E–H, M–R).

To determine if islet dysfunction promoted diabetes in *Pdx1-tTA/Smad7* mice, we measured serum and pancreatic levels of insulin and glucagon, and assessed islet phenotypes. Serum insulin concentration in *Pdx1-tTA/Smad7* mice was significantly reduced relative to littermate controls ( Figure 3C). By contrast, we did not detect significant differences in serum glucagon levels (48 ± 2 pg/ml in *Pdx1-tTA/Smad7* mice versus 55 ± 2 pg/ml in controls; *p* > 0.05), or numbers of glucagon ^+^ cells (1.16% ± 0.32% pancreas area in *Pdx1-tTA/Smad7* mice, *n* = 3; 0.74% ± 0.15% pancreas area in *Pdx1-tTA* controls, *n* = 3; *p* > 0.05). *Pdx1 ^+/−^* animals have lower serum insulin levels because of reductions in key glucose sensing and insulin secretion pathway components [ 24, 26, 27]. In bitransgenic animals, these defects were comparable to *Pdx1-tTA* controls and could not explain the more severe reduction in serum insulin or the development of overt diabetes (unpublished data). Therefore, we examined total pancreatic insulin content in *Pdx1-tTA/Smad7* mice following elimination of dietary doxycycline and found it to be markedly reduced ( Figure 3D). By contrast, wild-type and *Pdx1-tTA* mice had normal pancreatic insulin content ( Figure 3D). Compared to *Pdx1-tTA* controls, hyperglycemia, impaired glucose tolerance, and hypoinsulinemia were fully reversed in *Pdx1-tTA/Smad7* mice following re-administration of doxycycline ( Figure 3E–G). Moreover, total pancreatic insulin levels in *Pdx1-tTA/Smad7* mice following resumption of doxycycline administration were indistinguishable from those in control littermates (20.8 ± 0.8 ng/mg in *Pdx1-tTA/Smad7*; 17.7 ± 5.2 ng/mg in *Pdx1-tTA*; *n* = 3–5), and islets in bitransgenic *Pdx1-tTA/Smad7* on doxycycline appeared normal ( Figure 4A– 4D). Thus, Smad7-induced disruption of β cell function in *Pdx1-tTA/Smad7* mice was not permanent, and we postulate that islets in these mice responded to endogenous TGF-β signals that promptly re-established normal function. Collectively, our data provide the first evidence, to our knowledge, that disrupted islet TGF-β signaling may impair insulin production in adult islets, leading to pathogenesis of overt diabetes.

### TRE- *Smad7* Induction in Islets Reduces Expression of Characteristic β Cell Products

We next examined expression of β cell transcription factors known to govern insulin expression ( Figure 4). In vitro studies have established that the leucine-zipper transcription factor MafA is a direct regulator of insulin production in β cells [ 28– 31]. In islets of *Pdx1-tTA/Smad7* diabetic mice, *MafA* mRNA assessed by real-time PCR was reduced (0.72 ± 0.11, *Pdx1-tTA/Smad7*; 1.0 ± 0.07, *Pdx1-tTA*: *p* < 0.05; see Materials and Methods), and nuclear MafA in β cells was substantially decreased in islets from *Pdx1-tTA/Smad7* mice ( Figure 4E–G; *n* = 3). Thus, these data provide in vivo evidence that TGF-β signals may regulate MafA expression in mature β cells. Reduced MafA expression correlated with impaired islet and serum insulin levels in *Pdx1-tTA/Smad7* mice, consistent with recent evidence that MafA, in conjunction with other factors, can induce insulin expression in vivo [ 32].

In contrast, expression of other β cell transcription factors known to regulate insulin gene expression, like Nkx6.1, Pdx1, and NeuroD1, appeared unchanged ( Figure 4I– 4K, and unpublished data). In addition, we assessed KLF11 expression in these mice. KLF11 is a zinc-finger–containing transcriptional regulator of insulin expression, and recent studies showed that TGF-β ligand addition to cultured MIN6 β cells was sufficient to increase KLF11 mRNA levels [ 33]. Thus, Smad7 inhibition of TGF-β signaling might be expected to reduce KLF11 mRNA in *Pdx1-tTA/Smad7* mice. Using real-time RT-PCR, we did not detect a reduction in the level of KLF11 mRNA expression in islets from *Pdx1-tTA/Smad7* mice compared to *Pdx1-tTA* controls (3.3 ± 0.2 in *Pdx1-tTA/Smad7* versus 2.2 ± 0.3 in *Pdx1-tTA* controls). Thus, studies of Nkx6.1, Pdx1, NeuroD1, and KLF11 indicate that Smad7 expression in *Pdx1-tTA/Smad7* islets did not globally impair β cell functions.

Within 2 wk of doxycycline re-administration to diabetic *Pdx1-tTA/Smad7* mice, Smad7 expression was extinguished ( Figure 2K), islet morphology appeared normal, and MafA expression was again detectable in β cells ( Figure 4D and 4H). The reversal of these islet defects paralleled the resumption of normoglycemia, β cell phospho-Smad2 expression ( Figure 2N), and increased pancreatic insulin production in these mice ( Figure 3E–G). These results provide strong additional in vivo evidence that TGF-β signaling maintains the properties of differentiated islet β cells.

In addition to insulin and MafA, we asked whether Smad7 induction in *Pdx1-tTA/Smad7* mice disrupted expression of other characteristic β cell markers. Menin, the product of the *Men1* gene, and p27 ^Kip1^, the product of *Cdkn1b,* are expressed in mature β cells ( Figure 4I– 4J, 4M, and 4N). β cells proliferate in islets lacking menin or p27 ^Kip1^, leading to hyperplasia; thus, these β cell products are required to regulate β cell proliferation [ 34– 36]. The expression of both menin and p27 ^Kip1^ was nearly undetectable in islet β cells from all *Pdx1-tTA/Smad7* diabetic mice that were studied ( Figure 4K and 4O; *n* = 5), suggesting a mechanism for the mild islet hyperplasia observed in some animals ( Figure 4A– 4C; 2.5% ± 0.2% insulin ^+^ cell area in *Pdx1-tTA/Smad7* mice, *n* = 4; 1.5% ± 0.04% in *Pdx1-tTA* controls, *n* = 3; *p* < 0.05). Although we observed reduced menin and p27 ^Kip1^ expression in all *Pdx1-tTA/Smad7* diabetic mice that were tested, not all mice had detectable islet hyperplasia (unpublished data): thus, compensatory β cell growth in *Pdx1-tTA/Smad7* diabetic mice was heterogeneous, and additional studies are needed to elucidate the basis for islet growth in this model. Menin is a direct regulator of p27 ^Kip1^ in β cells [ 37, 38], and exposure of diabetic *Pdx1-tTA/Smad7* mice to doxycycline led to re-expression of p27 ^Kip1^ ( Figure 4L). Thus, Smad7 induction in *Pdx1-tTA/Smad7* mice inhibited expression of factors required to maintain characteristic β cell functions in adult pancreatic islets.

### Reduced Pancreatic MafA Expression in Gdf11-Deficient Embryos

**Figure 5:**
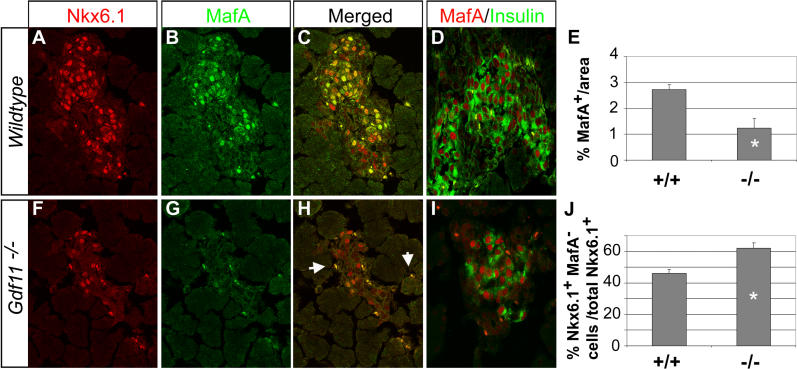
Reduced Expression of MafA by β Cells in Mice Lacking Gdf11 (A and B) Immunohistochemical detection of Nkx6.1 (red) and MafA (green) in wild-type embryos at E17.5. (C) Merge of (A) and (B). (D) Coexpression of insulin (green) and MafA (red) in wild-type embryos at E17.5. (E) Quantification of MafA ^+^ cells per unit area in *Gdf11* ^+/+^ and *Gdf11* ^−/−^ embryos. (F and G) Immunohistochemical detection of Nkx6.1 (red) and MafA (green) in *Gdf11* ^−/−^ embryos. (H) Merge of (F) and (G). (G–I) Reduction of MafA ^+^ cells in *Gdf11* ^−/−^ embryos. (J) Increased number of Nkx6.1 ^+^ MafA ^−^ cells in *Gdf11* ^−/−^ mice at E17.5. Asterisks in (E) and (J) indicate *p* < 0.05. Three to four animals per genotype were used for quantification. Arrows in (H) indicate autofluorescent blood cells. Original magnification of all images is 63×.

To test if loss-of-function mutations that impair TGF-β signaling can also disrupt terminal β cell differentiation, we examined mice lacking the TGF-β ligand Gdf11 ( Figure 5 and [ 39]). In *Gdf11* ^−/−^ mice, which die on P1, we recently showed that a subset of Nkx6.1 ^+^ cells in the embryonic pancreas failed to express insulin. This resulted in accumulation of anomalous Nkx6.1 ^+^ insulin ^−^ cells, and a greater than 50% reduction of pancreatic insulin ^+^ cell mass [ 3]. However, the basis for this defect in β cell maturation was not previously identified. Coinciding with the onset of insulin expression, embryonic Nkx6.1 ^+^ cells initiate expression of MafA [ 30], and by E17.5 we detected MafA expression in the majority of wild-type Nkx6.1 ^+^ cells ( Figure 5A–C and 5J). By contrast, MafA expression in Nkx6.1 ^+^ cells was impaired in *Gdf11* ^−/−^ mice ( Figure 5C, 5H, and 5J), resulting in a more than 50% reduction of MafA ^+^ cells in these animals ( Figure 5E– 5H). These findings suggest that impaired MafA expression is one reason for defective maturation and insulin expression by differentiating β cells in *Gdf11* ^−/−^ mice. Thus, similar to the effect of Smad7 misexpression, loss of Gdf11 reduced MafA expression in β cells. Collectively, these data suggest that TGF-β signaling is essential for expression of characteristic β cell functions in both the embryonic and adult pancreas.

## Discussion

These studies reveal a requirement for TGF-β signaling in maintaining pancreatic islet functions. Using an inducible system to express Smad7 conditionally in Pdx1 ^+^ cells, we disrupted TGF-β signaling in adult and nascent embryonic islets. One principal finding from this study is that Smad7 expression in adult pancreatic β cells leads to reduced expression of insulin and nuclear factors like MafA, menin, and p27 ^Kip1^. Expression of each of these factors in vivo is normally maintained in mature β cells [ 28, 29, 37, 38]. Smad7 expression may affect signaling pathways other than those regulated by TGF-β in specific in vitro contexts [ 40], but it has not been shown that Smad7 activity affects signaling other than TGF-β pathways in vivo. Moreover, we showed that levels of phosphorylated R-Smads were reduced in the adult islets and in embryonic pancreata expressing Smad7. Since TGF-β signaling is known to increase production of phosphorylated forms of R-Smad proteins, our data provide good evidence that TGF-β signaling is impaired by Smad7 induction in islets. In the developing pancreas, we found that loss of Gdf11, an endogenous TGF-β ligand required for β cell differentiation [ 3], led to impaired expression of MafA in subsets of nascent β cells. We conclude from these lines of evidence that TGF-β signaling is required in maturation and maintenance of pancreatic β cell functions.

### Disruption of TGF-β Signaling Impairs Expression of Hallmark β Cell Factors

Insulin expression is a defining feature of adult β cells; we showed that Smad7 expression led to severe reduction of total pancreatic insulin content. To begin to elucidate the mechanisms underlying insulin deficiency in these mice, we examined expression of known direct transcriptional regulators of insulin expression, and found evidence for reduction of MafA mRNA and nuclear protein. MafA is a member of the basic leucine zipper family of transcription factors, and several prior studies provide evidence that MafA is a direct regulator of insulin transcription [ 28– 32, 41]. Our results raise the possibility that reduced β cell expression of MafA leads to impaired insulin expression in bitransgenic *Pdx1tTA/Smad7* mice. While we measured a reduction of MafA mRNA in islets from these animals, the levels of immunostainable MafA protein in their β cells appeared more severely reduced. Recent studies have shown that *mafA* expression in β cells may be regulated by both transcriptional and post-transcriptional mechanisms. For example, covalent modifications can result in ubiquitin-mediated degradation of MafA protein [ 42]. Thus, multiple mechanisms may underlie the observed reduction in MafA following TGF-β signaling disruption in islets, and further studies are needed to address this point. Nevertheless, to our knowledge, these data provide the first demonstration of an extracellular signaling pathway that regulates MafA. Additional studies are needed to identify a mechanism for MafA reduction in islets after Smad7 misexpression.

Studies of mice with targeted disruptions of *MafA* suggest that insulin mRNA expression is reduced but that insulin protein levels are maintained in MafA-deficient islets [ 41]. Viewed in the context of prior studies of MafA function [ 28– 32], these are somewhat paradoxical findings; if correct, they suggest that the observed reduction of MafA expression in *Pdx1-tTA/Smad7* mice may not completely account for the severe decrease in total pancreatic insulin observed in these mice. If so, we speculate that targets of TGF-β signaling other than MafA in islets may function to ensure the maintenance of normal insulin production.

We detected expression of phospho-Smad2 in adult islets, providing evidence for active TGF-β signaling in islet cells. These results correlate well with prior results from our lab and others that demonstrated that TGF-β ligands, receptors, and R-Smads are expressed in adult islets [ 7, 8]. Smad7 expression led to reduced phospho-Smad2 levels in islets, consistent with the view that Smad7 disrupted TGF-β signaling in islets. In addition to direct interactions between Smad7 and R-Smads, our studies suggest that Smad7 expression may also impair TGF-β signaling indirectly through reduced expression of the nuclear factor menin. Prior studies have shown that menin directly interacts with nuclear R-Smads, functioning as a cofactor to control R-Smad DNA binding and transcriptional activity [ 43, 44]. Menin expression by β cells in *Pdx1tTA/Smad7* mice was clearly reduced; thus, we speculate that the absence of menin could enhance the inhibitory effects of Smad7 on downstream target gene expression.

### TGF-β Signals Are Necessary for Adult Islet Function

A second principal finding from this study is that Smad7 expression in β cells leads to conditional diabetes mellitus. Within 2–3 wk after Smad7 induction, *Pdx1-tTA/Smad7* mice developed overt diabetes, with elevated blood glucose levels, reduced glucose tolerance, and markedly decreased circulating insulin levels. Reductions of blood insulin were matched by corresponding reductions in total pancreatic insulin, suggesting that insulin production was defective in *Pdx1-tTA/Smad7* mice. These findings do not rule out the possibility that other islet β cell functions, including factors regulating stimulus-secretion coupling, may be disrupted by Smad7 induction and contribute to diabetes pathogenesis in *Pdx1-tTA/Smad7* mice: additional physiologic studies are needed to test this possibility. Re-exposure to doxycycline and loss of Smad7 led to rapid reversal of the diabetes in *Pdx1-tTA/Smad7* mice, suggesting that defective islets in these animals remain responsive to endogenous restorative signals. Phospho-Smad2 expression in islets resumed following cessation of Smad7 expression, making it likely that one or more endogenous TGF-β ligand(s) promote this restoration of islet function.

These observations also complement prior studies of TGF-β signaling in the adult endocrine pancreas. Previously we described that adult mice harboring trans-heterozygous null alleles in the activin receptor IIA and activin receptor IIB genes have impaired glucose tolerance, reduced β cell mass, and reduced circulating insulin levels [ 2]. Likewise, expression of dominant-negative type II receptor transgenes in β cells led to hypoinsulinemia and impaired glucose tolerance [ 14]. In neither prior study, however, did these genetic changes progress to overt diabetes, suggesting the possibility of redundant TGF-β signaling activities to maintain islet function. Moreover, it remained unclear if the modest physiologic and morphologic phenotypes observed in this prior work reflected a requirement for TGF-β in maintenance of adult islet functions, or instead resulted from defects in islet differentiation during embryonic pancreas development [ 2, 3]. Our ability to induce Smad7 expression specifically in postnatal *Pdx1-tTA/Smad7* mice therefore provided a unique opportunity to elucidate the roles of TGF-β signaling in adult islets. Thus, multiple experimental approaches used in prior studies and the work presented here provide strong evidence that TGF-β signaling is required to maintain adult islet functions essential for appropriate in vivo glucose regulation. Since Smad7 blocks multiple receptor-activated TGF-β signaling pathways, our work has not yet revealed roles of individual receptor-mediated TGF-β pathways in maintaining adult islet functions.

### Conditional Disruption of TGF-β Signaling Impairs Pancreas Development

Smad7 misexpression in the developing pancreas disrupted morphogenesis and islet differentiation. These observations fit well with previous studies revealing disrupted pancreas development in embryos with defects in TGF-β signaling [ 1– 3]. For example, deficiency for the ActRIIB receptor led to malformation of multiple foregut organs including the spleen, pancreas, and stomach, and modest islet β cell hypoplasia [ 2]. Gdf11, a TGF-β ligand, can bind to ActRIIB to activate Smad2 and Smad4 [ 5]. In mice lacking *Gdf11* or *Smad2,* islet defects similar to those in ActRIIB mutants, including β cell hypoplasia and impaired insulin synthesis, were observed [ 3]. These studies of oligogenic mutant combinations suggested that TGF-β signaling was required for β cell differentiation, and raised the possibility that redundant TGF-β signaling activities regulated β cell fate. Thus, we postulated that misexpression of a potent TGF-β signaling inhibitor like Smad7, which is capable of blocking signaling by TGF-β, activin, BMPs, and corresponding R-Smads, would generate much more severe β cell hypoplasia than observed in these prior studies. We observed a severe reduction of phospho-Smad2/3 and phospho-Smad1/5/8 expression, and loss of β cells in bitransgenic *Pdx1-tTA/Smad7* embryos. Thus, Smad7 expression was sufficient to disrupt TGF-β signaling significantly, resulting in a nearly complete absence of insulin ^+^ β cells. We speculate that this method for conditional Smad7 expression may be useful for elucidating TGF-β roles in other organs and tissues.

Near-complete β cell loss precluded detailed analysis of the fate of β cells in bitransgenic *Pdx1-tTA/Smad7* embryos. To study the basis for reduction of insulin ^+^ cells in mice with disrupted TGF-β signaling, we therefore analyzed *Gdf11*-deficient embryos, which displayed defects in pancreatic islet cell differentiation similar (although qualitatively less severe) to those in *Pdx1-tTA/Smad7* mice. In *Gdf11* ^− */*−^ animals, we observed the failure of Nkx6.1 ^+^ cells to express MafA, a step known to presage the onset of insulin expression by β cell progenitors during islet formation [ 30] Thus, TGF-β signaling appears to be essential for β cell maturation in pancreas development.

In conclusion, our data support the hypothesis that TGF-β signaling is required to establish and maintain hallmark functions of mature β cells in pancreatic islets. Reversal of diabetes and resumption of normal β cell function in our studies of conditional TGF-β signaling disruption suggests that dysfunctional islets in diabetic animals may remain responsive to restorative signals. Identification of endogenous TGF-β ligands, signal transduction components, and targets of TGF-β signaling in islets therefore merits further study. Dissection of pathways that establish and maintain defining properties of β cells will be useful for creating cell-based strategies to treat pancreatic endocrine disorders like diabetes mellitus.

## Materials and Methods

### Generation, breeding, and genotyping of mice

Plasmid TRE-Smad7 was constructed by inserting a 3.0-kb EcoRI-BamHI PCR clone of human Smad7 (hSmad7) [ 21] downstream of TRE, which consists of seven copies of the tet operator and a minimal CMV promoter (Clontech Laboratories, Palo Alto, California, United States). After gel purification, the TRE-Smad7 DNA fragment was microinjected into the pronuclei of fertilized mouse eggs in the Stanford University Transgenic Core Facility. The injected embryos were cultured in vitro to the two-cell stage and transferred into the oviducts of 0.5-d pseudopregnant female mice. Mice were genotyped for the presence of the transgenes by PCR using the following forward (F) and reverse (R) primers: F = CCCAATGACCACGAGTTTATGC; and R = CTGACAAGTGAAATGATGACCGC. The PCR reaction for TRE-Smad7 was performed by denaturation at 95 °C for 10 min and 35 cycles of amplification (95 °C for 45 s, 62 °C for 60 s, and 72 °C for 90 s), followed by a 10-min extension period at 72 °C. From 20 independent lines, three TRE-Smad7 founders transmitted the integrated transgene as assessed by PCR and Southern analyses, and had no detectable ectopic Smad7 expression. One of these TRE-Smad7 mouse lines, line C, was used in these studies. Doxycycline (1 g/l) was administrated orally in the drinking water and replaced three times per week. Targeted disruption and genotyping of Gdf11 [ 3, 39] and the construction and genotyping of Pdx1-tTA mouse strains have already been described [ 22]. Genomic DNA was prepared from embryonic heads or yolk sacs and adult tails as previously described [ 45]. RIP-rtTA animals were obtained from the Joslin Diabetes Center (gift from Drs. D. Mathis and C. Benoiste, Boston, Massachusetts, United States). Genotyping protocols and primers were the same as for the Pdx1-tTA animals. Pdx1-tTA animals were maintained on a C57BL/6J background. Gdf11 ^+/−^ and RIP-rtTA animals were maintained on a mixed 129/Sv-C57BL/6J hybrid genetic background.

### Morphometric and histologic methods

Point-counting morphometry, hormone-positive area, and adult islet area were performed using standard morphometric techniques [ 2, 3]. The entire pancreas of both embryonic and adult mice was serially sectioned, and 11–25 independent sections per pancreas were stained and counted. To obtain representative results, all quantification of immunostaining was performed by counting numbers of positive-stained cells and dividing by the area of total pancreatic tissue. Data were collected from at least three animals per genotype. All data represent the average of the indicated number of animals ± the standard error of the mean (SEM). Two-tailed *t* tests were conducted to determine statistical significance. Unless otherwise noted, all immunofluorescence presented here was performed on cryogenic sections. Cryogenic sections were prepared as follows: following brief fixation in 4% paraformaldehyde, tissue was soaked with 30% sucrose overnight, embedded in O.C.T. compound (Tissue-Tek; Electron Microscopy Sciences, Hatfield, Pennsylvania, United States) and frozen. Microtomy of paraffin and frozen tissues resulted in sections 7–10 μm thick. Immunohistochemistry, immunofluorescence, and localization of antigens using paraffin sections were performed with methods previously described [ 2]. Immunofluorescent pictures were captured using confocal microscopy. Antigen retrieval (Vector Laboratories, Burlingame, California, United States) was used on all paraffin and cryogenic sections stained with antisera for nuclear factors except for menin. The primary antisera used were: guinea pig anti-glucagon (1:100; Linco, St. Charles, Missouri, United States), mouse anti-glucagon (1:500; Sigma-Aldrich, St Louis, Missouri, United States), guinea pig anti-insulin (1:100; Linco, or 1:250; Abcam, Cambridge, United Kingdom), mouse anti-Ki67 (1:200; BD Pharmingen, San Diego, California, United States), rabbit anti-MafA (1:500; Bethyl Laboratories, Montgomery, Texas, United States) rabbit anti-Menin (1:200; Santa Cruz Biotechnologies, Santa Cruz, California, United States), guinea pig anti-Nkx6.1 (1:1000; M. Sander, University of California at Irvine, Irvine, California, United States), rabbit anti-Pdx1 (1:200; C. Wright, Vanderbilt University, Nashville, Tennessee, United States, or 1:500; Chemicon, Temecula, California, United States), rabbit anti-phospho Smad1/5/8, phospho-Smad2 and phospho-Smad2/3 (1:50; Chemicon), mouse anti-p27 ^Kip1^ (1:50; Pharmingen), goat anti-Smad7 (1:100; Santa Cruz Biotechnologies). Secondary antibodies (Jackson ImmunoResearch, West Grove, Pennsylvania, United States) for immunofluorescent or peroxidase detection of primary antibodies were respective animal Ig conjuguated to Cy3 (1:400), FITC (1:200), or biotin (1:200). MafA was detected using a biotinylated secondary antibody followed by streptavidin-Cy3 or FITC (1:1000 or 1:200; Jackson ImmunoResearch). Tissues for anti-menin immunostaining were treated with 0.1% pepsin in 0.1% HCl for 5 min to expose the antigen. Immunoperoxidase detection for insulin and p27 ^Kip1^ antibodies was performed using Vectastain Elite ABC and DAB kits (Vector Laboratories).

### Islet isolation and PCR methods

Islets were isolated and RNA prepared using previously described methods [ 46]. Genomic DNA was removed using 1 μl of RNase-free DNase-I (Boehringer, Ingelheim, Germany) per 1.5 μg RNA. cDNAs were synthesized as previously described [ 2]. RT-PCR for human Smad7 transcript was performed on standardized amounts of cDNA using the following forward (F) and reverse (R) primers: F = GCTTGCTGGCCTAATAGCAG; and R = GCAGGACACCCTGATAGGAA. These primers are predicted to give a 697-bp band and were designed to span the first intron of hSmad7. The RT-PCR band of this size was gel-isolated with a commercial kit (Qiagen, Valencia, California, United States) and sequenced (Sequetech, Mountain View, California, United States) to confirm the specificity of the PCR. Real-time RT-PCR was performed using a 7300 Real Time PCR System from Applied Biosystems (ABI, Foster City, California, United States) on cDNA made from islets isolated from three animals per genotype to ensure adequate RNA isolation. Real-time RT-PCR on islets was performed in triplicate for each condition and results presented as the mean ± SEM. MafA and KLF11 real-time RT-PCR were quantified relative to a β-actin internal control. Labeled primers were from ABI.

### Animal care and metabolic studies

Animals were maintained on a standard light/dark cycle and handled in accordance with Stanford University Animal Care and Use guidelines. Smad7 expression in Pdx1-tTA/Smad7 embryos was inhibited by providing pregnant females doxycycline (1 g/l) in their drinking water. To permit Smad7 expression during embryogenesis, pregnant females were maintained without doxycycline throughout pregnancy, and litters were collected at specified times. To induce Smad7 in Pdx1-tTA/Smad7 adults, animals born to doxycycline-fed mothers were maintained on doxycycline until 3 wk of age. Animals were then taken off doxycycline and subjected to physiologic testing (see text). In some Pdx1-tTA/Smad7 mice and age-matched littermates taken off doxycycline at 3–5 wk of age, doxycycline administration (1 g/l) was resumed at 7–9 wk of age. Animals were subjected to physiologic tests and then sacrificed at 5 mo of age for total pancreatic insulin analysis. All data collected were from at least three animals per genotype. In contrast to the tTA gene product expressed from the Pdx1 locus, which is inhibited by doxycycline, the rtTA protein expressed from RIP *cis*-regulatory elements in the RIP-rtTA transgene is activated by doxycycline. Thus, to induce Smad7 expression in RIP-rtTA/Smad7 animals, animals were given doxycycline (1 g/l) in their drinking water at 3 wk of age and maintained on doxycycline for 7 wk. Blood glucose concentration and glucose tolerance tests were measured weekly, as described [ 2, 47]. Serum insulin concentrations were obtained either from mouse tail bleeds or from the hearts of anesthetized mice immediately prior to sacrifice [ 47]. Pancreatic insulin content was measured after the pancreas was homogenized in 70% acid-ethanol [ 24]. To allow comparison of pancreatic insulin content in different groups of mice, insulin levels were normalized to pancreatic protein content or weight. Both serum insulin and total pancreatic insulin concentrations were determined using the Mercodia Ultrasensitive Mouse Insulin ELISA kit (Alpco Diagnostics, Windham, New Hampshire, United States). Glucagon concentration was determined by radioimmunoassay (Linco).

## Supporting Information

Figure S1 Smad7 Expression Impairs TGF-β Signaling and Disrupts Embryonic Pancreas Development (A–D) Immunostaining with antiserum that detects phosphorylated Smad1, Smad5, and Smad8 (“p-Smad1/5/8” [D–E]) or phosphorylated Smad2 and Smad3 (“p-Smad2/3” [F–G]) in pancreatic epithelia at P1 in mice with the indicated genotype. Compared to wild-type controls, expression of p-Smad1/5/8 and p-Smad2/3 is clearly reduced in bitransgenic *Pdx1-tTA/Smad7* mice.
(E–H) Immunostaining with reagents to detect apoptotic TdT-mediated dUTP nick-end labeling ^+^ nuclei (E–F, arrows) or replicating Ki67 ^+^ cells (G–H, white nuclei) in pancreatic epithelia at E15.5 is indistinguishable in wild-type and bitransgenic *Pdx1-tTA/Smad7* mice. Original magnification is 20× for all micrographs.
(I) Defects in numbers of insulin ^+^ or glucagon ^+^ cells was revealed by immunostaining and morphometry. % cells/area was measured as described in Materials and Methods on three animals per genotype. * *p* < 0.05.
(1.5 MB JPG).

## Abbreviations

BMP: bone morphogenetic protein
E[number]: embryonic day [number]
Gdf11: growth/differentiation factor 11
P[number]: postnatal day [number]
phospho-Smad2: phosphorylated Smad2
R-Smad: receptor-regulated Smad
RIP: rat insulin promoter
rtTA: reverse tetracycline transactivator
TGF-β: transforming growth factor-β
TRE: tetracycline response element
tTA: tetracycline-regulated transactivator
